# 903. A Real-world Study Assessing the Risk of Lipid Changes and Other Metabolic Effects Associated with Integrase Inhibitor-based Antiretroviral Therapy

**DOI:** 10.1093/ofid/ofab466.1098

**Published:** 2021-12-04

**Authors:** Zachary Gruss, Tyler Stone, James Johnson, Caryn Morse, John Williamson

**Affiliations:** Wake Forest Baptist Health, Winston-Salem, North Carolina

## Abstract

**Background:**

Integrase inhibitor (INSTI)-based antiretroviral therapies (ART) are first-line for HIV infection. Recent studies have identified metabolic complications of INSTI regimens, especially when TAF is included. The objective of this study was to assess lipid changes associated with INSTI-based ART.

**Methods:**

This was a retrospective, observational, single-center study. Patients age ≥ 18 with HIV infection, receiving care Jan 2004 to Jul 2019, and prescribed the same ART for ≥ 6 consecutive months were randomly selected from a computer-generated list. Unique ART regimens prescribed per patient were considered “exposures”. Patients were followed up to 2 years per exposure. INSTI-based exposures included an INSTI plus one or more NRTI. Non-INSTI-based exposures included a PI or NNRTI plus one or more NRTI. Lipid panel results were recorded before and during exposures. Lipid changes with INSTI and non-INSTI exposures were compared. Other metabolic outcomes were recorded.

**Results:**

192 exposures were identified (99 INSTI, 93 non-INSTI) among 132 patients. The majority were Black (69.3%) and male (61.9%). Mean age and BMI were 42.0 ± 11.3 and 26.2 ± 5.5 kg/m^2^, respectively. 72 (37.5%) were ART-naïve. 53.5% INSTI recipients received BIC or DTG. Hypertriglyceridemia (HTG) occurred more often during INSTI exposures (13.1% vs 4.3%; p = 0.031). Mean change in TG concentration was +10.1 and -15.0 mg/dL for INSTI and non-INSTI regimens, respectively (p = 0.105). Patients receiving INSTIs were more commonly prescribed a new lipid-lowering therapy (LLT) or an increase in dose of preexisting LLT (14.1% vs 2.2%; p = 0.003). Of those that received an INSTI-regimen and either TAF, TDF, or no tenofovir, 17.4%, 10.3%, and 8.3% developed HTG, respectively (p = 0.49). There was no difference between groups in HDL or LDL changes. Among INSTI and non-INSTI groups, weight increased by +3.8 and +2.1 kg, respectively (p = 0.129). Mean weight change was less in INSTI recipients whose regimen included TDF (+1.4 vs +4.8 kg; p = 0.053).

**Conclusion:**

Treatment with INSTI-based ART was associated with HTG. LLTs were utilized more often among INSTI recipients, and this limited our ability to fully characterize lipid changes in this real-world study. Weight gain among INSTI recipients may be attenuated if TDF is included in the regimen.

**Disclosures:**

**James Johnson, PharmD**, **FLGT** (Shareholder) **Caryn Morse, MD**, **Chimerix** (Scientific Research Study Investigator)**Covis Pharma** (Scientific Research Study Investigator)**Gilead Sciences Inc.** (Scientific Research Study Investigator)**Ridgeback Biotherapeutics** (Scientific Research Study Investigator)**Roche** (Scientific Research Study Investigator)**SCYNEXIS, Inc.** (Scientific Research Study Investigator)**Theratechnologies** (Advisor or Review Panel member)**Viiv** (Advisor or Review Panel member)

